# Role of Fe, Transferrin and Transferrin Receptor in Anti-Tumor Effect of Vitamin C

**DOI:** 10.3390/cancers14184507

**Published:** 2022-09-17

**Authors:** Jia Qiu, Renbo Wu, Yali Long, Lei Peng, Tianhong Yang, Bing Zhang, Xinchong Shi, Jianbo Liu, Xiangsong Zhang

**Affiliations:** Department of Nuclear Medicine, The First Affiliated Hospital of Sun Yat-Sen University, Zhongshan Er Road, Guangzhou 510030, China

**Keywords:** vitamin C, iron content, transferrin/transferrin receptor, PET-CT

## Abstract

**Simple Summary:**

High-dose vitamin C (VC) inhibits cell proliferation in a variety of tumors, which is mediated by ROS. Iron is an important factor in the Fenton reaction, in which H_2_O_2_ reacts with ferrous iron to produce hydroxyl radicals, which may enhance the killing of tumor cells. In this study, we investigated the roles of iron level and the mainly pathway of iron uptake-TF/TFR system on VC-induced cytotoxicity. We conducted a preliminary evaluation of the potential value of ^68^Ga-citrate imaging in the anti-tumor therapy of VC combined with iron supplementation.

**Abstract:**

High-dose vitamin C (VC) exhibits anti-tumor effects, and the cytotoxicity of VC is correlated with oxidative stress. However, iron, as a redox metal, plays an important effect in redox cycling and free radical formation in cells. This study addresses the role of iron ion in the cytotoxicity of VC. We found that iron supplementation increases the anti-tumor effect of VC, which was influenced by the cellular iron uptake pathway–transferrin (TF)/transferrin receptor (TFR) system. The TFR expression of tumors can be assessed by ^68^Ga-citrate PET imaging, and it would be helpful to screen out the tumor type which is more sensitive to VC combined with an iron supplementation treatment.

## 1. Introduction

Increasing attention has been paid to the anti-tumor effect of vitamin C (VC). High-dose VC (also known as pharmacological doses of VC) inhibit various forms of tumor proliferation, which have been confirmed in many cell and animal model experiments [1,2]. Although the anti-tumor mechanisms of VC remain unknown, the evidence increasingly shows that the cytotoxicity of VC is dependent on the generation of hydrogen peroxide (H_2_O_2_) [2,3]. Because of the low expression of catalase in tumor cells, the ability to clear H_2_O_2_ in tumor tissue is weaker than in normal tissue. Thus, the sensitivity of tumor cells to toxicity induced by H_2_O_2_ may help VC to be a specificity treatment strategy as an anti-tumor drug [2,4]. Previous research confirmed that the formation of H_2_O_2_ depends on the oxidation of VC, which is related to metal ion redox chemistry [5,6].

Iron is an essential nutrient that is involved in the various functions of cells, including oxygen transport, cell respiration and DNA synthesis [7]. Iron in the human body requires a dynamic balance; iron deficiency leads to cell growth arrest, while excess iron leads to cytotoxicity, causing cell damage. However, iron induces DNA strand breaks, which lead to more aggressive tumor invasiveness [7,8]. Iron is involved in oxidation–reduction reactions; thus, it takes part in free radical generating reactions, which are mediated by the Fenton reaction. In the Fenton reaction, H_2_O_2_ is donated an electron from ferrous iron and then produces the hydroxyl radical, a highly reactive oxygen species (ROS), which may enhance the killing of tumor cells [9]. Many iron-targeted tumor treatment strategies have recently come under investigation [10]. 

The transferrin (TF)/transferrin receptor (TFR) system is the main pathway of cellular iron uptake. Circulating iron is complexed in TF; then, TF binds to the TFR in the surface of cancer cells [11]. Many kinds of cancer cells have a higher level of iron than normal cells due to the highly expressed TFR on the surface of tumor cells, such as breast cancer and colon cancer [12,13,14]. Gallium (Ga) is an iron analogue, which could bind to circulating TF and uptake by cells via TFR on the cell surface [15]. Moreover, gallium activates the lactoferrin in neutrophils. Thus, ^67^Ga-citrate, as a classical nuclear medicine imaging agent, is often used in the clinical evaluation of inflammation [16]. ^68^Ga is an isotope of ^67^Ga, and they have similar chemical and physiological behaviors [17]. The application of ^68^Ga-citrate to tumor research has drawn increasing consideration based on the high TFR expression of malignant tumor cells [18]. 

Based on the research cited above, we hypothesized that intracellular iron content will affect cytotoxicity induced by VC. In this study, we observed the inhibitory effect of VC to tumor cell proliferation. We investigated the roles of iron levels and the main pathway of the iron uptake–TF/TFR system on VC-induced cytotoxicity. In animal experiments, we explored the connection between ^68^Ga-citrate positron emission tomography imaging (PET-CT) and the TFR expression of tumors. We also conducted a preliminary evaluation of the potential value of ^68^Ga-citrate imaging in the anti-tumor therapy of VC combined with iron supplementation.

## 2. Materials and Methods

### 2.1. Cell Culture and Reagents

Human prostate cancer DU145 and PC3 cell lines, human colorectal cancer HCT8, HCT116, HT29, RKO cell lines, murine glioma C6 cell lines and murine colorectal cancer CT26 cell lines were obtained from the Chinese Academy of Sciences Cell Bank. RKO cells were cultured in DMEM medium, and other cell lines were cultured in RPMI-1640 medium (Gibco, Carlsbad, CA, USA), supplemented with 10% fetal bovine serum (Gibco, Carlsbad, CA, USA) and 1% penicillin/streptomycin (MCE, Monmouth Junction, NJ, USA). The cells were grown in a humidified atmosphere of 5% CO_2_ at 37 °C. L-ascorbic acid (Alfa Aesar, Shanghai, China), Holo-TF (Sigma, St. Louis, MO, USA), apo-TF (Sigma, St. Louis, MO, USA), FAS (Sigma, St. Louis, MO, USA), deferoxamine (DFO) (Sigma, St. Louis, MO, USA) and diethyldithiocarbamate (DDC) (Sigma, St. Louis, MO, USA) were dissolved in physiological saline solution and neutralized with sodium hydroxide before use. Diethylenetriaminepentaacetic acid (DTPA) (Sigma, St. Louis, MO, USA) was dissolved in a sodium hydroxide solution and neutralized with hydrochloric acid before use.

### 2.2. Cell Proliferation Assay 

Cell viability assays were carried out using a Cell Count Kit-8 following the manufacturer’s instructions (Dojindo, Kumamoto, Japan). Briefly, the PC3, DU145, HCT8, HCT116, HT29, RKO, C6 and CT26 were pre-cultured in 96-well plates with a density of 8 × 10^3^, 6 × 10^3^, 10 × 10^3^, 12 × 10^3^, 6.5 × 10^3^, 16 × 10^3^, 10 × 10^3^ and 10 × 10^3^ cells/well, respectively, for 12 h. We then added different doses of VC (0 mM, 1 mM, 2 mM, 4 mM and 8 mM) and incubated the cells for 6 h. Each well was supplemented with 100 µL WST-8 solution and then incubated for 2 h. Absorbance was measured at 450 nm using a Multiskan FC photometer (Thermo Fisher Scientific, Waltham, MA, USA).

To test the different effect of intracellular iron and extracellular iron on VC-induced cytotoxicity, in the iron supplementation experiment, cancer cells were pre-incubated for 3 h with 10 μM holo-TF or FAS and washed with PBS prior to different doses of VC treatment or holo-TF or FAS and VC exposure simultaneously in order to provide conditions under which the iron supplementation was either intracellular or extracellular during VC exposure. In the iron chelator experiment, we used a combination drug of 200 μM DFO and 1 mM DTPA for 3 h. The dose of DDC was 10 μM.

### 2.3. Analysis of Reactive Oxygen Species 

The PC3 cells were seeded in a 12-well microplate (7.0 × 10^3^ cells/well and 9.0 × 10^3^ cells/well). The cells were washed three times with Hank’s Balanced Salt Solution (HBSS) and incubated with 10 μM H2DCF-DA (GeneCopoeia Rockville, MD, USA) for 20 min. The cells were then washed three times with the HBSS to remove any free dye. Thereafter, the cells were exposed to 10 μM holo-TF for 60 min and then to 4 mM VC. To visualize the DCF-DA, fluorescence was detected at 488 nm excitation using a fluorescence microscope (Leica DMI8, Nussloch, Germany).

### 2.4. Flow Cytometry

Cell apoptosis was assessed by flow cytometry with Annexin V-FITC/PI apoptosis kit (BD Pharmingen, San Diego, CA, USA). After being pre-cultured for 12 h, cells (1.8 × 10^5^ cells/well for the DU145 cells or 2 × 10^5^ cells/well for the PC3 cells in 6-well plates) were incubated with 6 mM VC for 16 h at 37 °C or pre-incubated with ROS scavenger- NAC (N-acetyl-L-cysteine) for 1 h before VC treatment. The cells were harvested using a 0.05% trypsin solution without ethylene diamine tetraacetic acid (EDTA) and washed twice with phosphate buffer saline (PBS). The cells were stained with fluorescein isothiocyanate-labeled Annexin V and counterstained with propidium iodide, which was followed by resuspension in the binding solution. Apoptotic cells were determined using a CytoFLEX S cytometer (Beckman Coulter, Fullerton, CA, USA).

### 2.5. Cytological Analysis of Catalytic Ferrous Iron

FeRhonox-1 (Goryo Chemical, Sapporo, Japan) was stored at −20 °C and dissolved in high-purity dimethyl sulfoxide (DMSO) to produce a 1 mM solution, which was further diluted (1:100) with serum-free medium to a working concentration of 10 μM. The DU145 and PC3 cells were seeded in a 24-well plate (4 × 10^5^ cells/well and 4.5 × 10^5^ cells/well) and treated with 10 μM holo-TF or FAS with or without apo-TF for 60 min in the dark. The cells were washed with Hank’s Balanced Salt Solution (HBSS) and incubated with 10 μM FeRhoNox-1 solution for 60 min. Then, the cells were washed in HBSS three times to remove any free dye. Finally, the cells were observed with a fluorescence microscope (Leica DMI8, Leica, Wetzlar, Germany). 

### 2.6. siRNAs and Transfection

The DU145 and PC3 cells were seeded in a 6-well plate with about 60% confluent density the day before transfection. The next day, cells were transfected. According to the manufacturer’s protocol, siRNA (50 nM) (HeMa, Huzhou, ZheJiang, China) and Lipofectamine RNAimax reagent (5 μL/well) (Invitrogen, Carlsbad, CA, USA) were prepared as instructed in OptiMEM transfection media (250 μL/well) (Gibco, Carlsbad, CA, USA). The cells continued to be used for different experiments after 48 h, such as the CCK8 and Western blot. The siRNA sequences were as follows: TFR siRNA-1: CACACUCAGUUUCCAUCUTT; TFR siRNA-2: CCACUGUUGUAUACGCUUAUUTT; TFR siRNA-3: UGAGUUUACAGUGGCUGUAUUTT. After 48 h of transfection, a Western blot was used to confirm the knockdown effect of TFR. 

### 2.7. Western Blot Assay

The cells (1 × 10^6^ cells) were lysed using RIPA (CWBIO, Peking, China). Protein concentrations were determined using a Micro BCA protein assay kit (CWBIO, Peking, China) following the manufacturer’s instructions. The proteins (approximately 30 µg each lane) were separated with 10% sodium dodecyl sulfate polyacrylamide gel electrophoresis (SDS-PAGE) and then transferred onto a polyvinylidene fluoride (PVDF) membrane. The PVDF membranes were blocked for 1 h with 5% non-fat milk at room temperature and subsequently incubated with anti-TFR antibody (ab84036, 1:1000; Abcam, Cambridge, UK) and anti-β-actin (4970, 1:2000; Cell Signaling Technology, Beverly, MA, USA) at 4 °C overnight, which was followed by secondary antibodies (7074, 1:2000; Cell Signaling Technology, Beverly, MA, USA) at room temperature for 1 h. The membranes were visualized using a chemiluminescence imaging analysis system (Image Quant Las4000 mini, GE Healthcare, Uppsala, Sweden).

### 2.8. Cellular Uptake Experiment In Vitro

Cellular uptake was performed using prostate cancer PC3 cells. For the in vitro experiments, cells were plated in 12-well plates (1.0 × 10^4^ cells/well). After 24 h of cultivation, the medium was replaced by 1 mL DPBS containing 2 μCi ^68^Ga-labeled citrate and incubated for 60 min at 37 °C. Incubation was then stopped by removing the medium and washing the cells thrice with cold DPBS. Subsequently, cells were lysed with 0.3 mL of 1 mol/L NaOH and radioactivity was measured with a gamma counter and calculated as percent applied dose/protein concentration.

### 2.9. Prostate Cancer Xenograft Tumor Models

All animal experiments were approved by the Institutional Animal Care and Use Committee of the First Affiliated Hospital of Sun Yat-sen University and conformed to the National Institutes of Health Guidelines for the Care and Use of Laboratory Animals. Stably transfected PC3 cell lines were obtained from FengHui Biotechnology (Changsha, Hunan, China) and were verified by qPCR and Western blotting. The siRNA sequences were as follows: TFR siRNA: CCACUGUUGUAUACGCUUAUUTT. Six-week-old male BALB/c nude mice, purchased from Jicui Yaokang Biotechnology (Nanjing, Jiangsu, China) were subcutaneously injected with PC3 cells (5.0 × 10^6^) in 100 μL PBS at the bilateral flank region. The tumors were allowed to grow to the size of 50~100 mm^3^; then, a ^68^Ga-citrate PET-CT scan was performed.

### 2.10. PET-CT Studies and Image Analysis 

^68^Ga-citrate was produced by a ^68^Ge/^68^Ga generator. The PET-CT images of tumor-bearing mice were obtained by a GE Discovery MI PET/CT scanner (GE Healthcare, Milwaukee, MI, USA) with AW workstation and a Micro-PET scanner (Siemens, Erlangen, Germany) equipped with Inveon Research Workplace 4.1 software. Before the scan, all mice were anesthetized and injected with 3.7 MBq (100 Ci) ^68^Ga-citrate. PET imaging acquisition was conducted 60 min after injection. After the image acquisition, attenuation correction was performed using the workstation-specific software of PET-CT, and then, the images were reconstructed to obtain images of three cross-sections: transverse, sagittal and coronal. The regions of interest (ROIs) of tumors and thigh muscle tissue areas were delineated in the reconstructed images. Finally, data results were calculated as the tumor uptake target to normal tissue ratio (TNR).

### 2.11. Immunohistochemistry 

To block endogenous peroxidase activity, we cut tumor tissues into 4 mm thick slices and incubated them with 3% hydrogen peroxide for 25 min at room temperature. Thereafter, the tissues were incubated with bovine serum albumin (BSA) for 30 min at room temperature to block nonspecific binding. The sections were incubated with primary antibodies (TFR, ab84036, 1:500; Abcam, Cambridge, UK) overnight at 4 °C and then incubated with a biotinylated secondary antibody (HRP-labeled goat anti-rabbit IgG, 1:200, Cell Signaling Technology, MA, USA) for 50 min at room temperature. The immunoreactions were visualized using a Dolichos Biflorus Agglutinin chromogenic reagent kit, which was followed by hematoxylin staining for 3 min. The sections were imaged using a fluorescence microscope (Leica DMI8) and analyzed with ImageJ (National Institutes of Health, Bethesda, MD, USA).

### 2.12. Statistical Analysis

All the statistical analyses were performed using SPSS 22.0 software (SPSS Inc., Chicago, IL, USA) and GraphPad Prism (GraphPad Software, GraphPad Software, San Diego, CA, USA). The significance of differences between groups was determined by a two-tailed Student’s t-test. The statistically significant differences between experimental groups were set to *p* < 0.05 (* p < 0.05; ** p <0.01; *** p <0.001). 

## 3. Results

### 3.1. The Anti-Tumor Effect of High-Dose VC Is Mediated by ROS Generation

**Figure 1:**
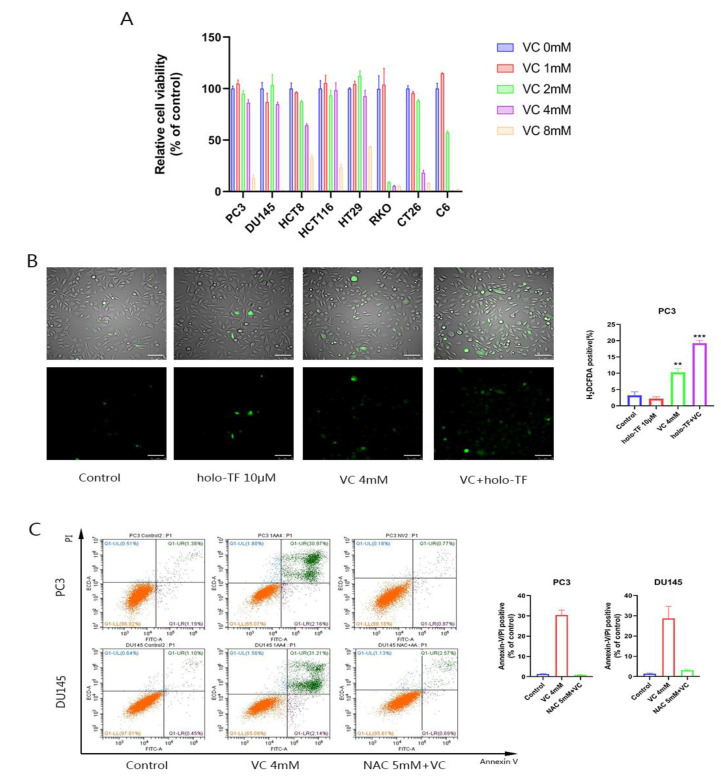
High-dose VC inhibits the proliferation of eight kinds of cancer cells, which are mediated by ROS generation. (**A**) Relative cell viability of eight kinds of cancer cells after different doses of VC treatment for 6 h. (**B**) Merge photos of ROS production after 4 mM VC and/or 10 μM holo-TF treatment for 30 min in PC3. The ROS production was assessed using fluorescence microscopy. Scale bars: 100 μm. (**C**) PC3 and DU145 cell apoptosis evaluated by an annexin-V/PI assay after treatment with 4 mM VC for 16 h alone and/or pretreatment with 5 mM NAC for 1 h, and then with VC treatment. ** *p* < 0.01, *** *p* < 0.001.

High-dose VC suppresses the proliferation of eight cancerous cell lines (PC3, DU145, HCT8, HCT116, HT29, RKO, C6 and CT26) (Figure 1A). Through observation via a fluorescence microscope, we found that VC could induce ROS generation in prostate cancer cells (Figure 1B). The apoptosis assay results showed that the pre-incubated NAC had remarkable apoptotic inhibition in DU145 and PC3 cells compared to VC treatment alone, as demonstrated by decreased Annexin V/PI positive cells (Figure 1C). The cytotoxicity caused by the VC treatment alone could be reversed after pretreatment with the NAC antioxidant for 1 h. Our apoptotic assays showed that a single treatment with VC or NAC pre-therapy before 4 mM VC treatment induced 30.43% or 0.90% non-viable apoptotic cells in PC3 cells, and 28.71% or 3.13% non-viable apoptotic cells in DU145 cells, respectively. These findings suggest that ROS generation mediates VC-induced apoptosis in prostate cancer cells.

### 3.2. Iron Supplementation with the Addition of Holo-TF and FAS Enhanced Intracellular Iron Levels; the Iron Uptake of Cancer Cells Is Dependent on the TF/TFR System

**Figure 2:**
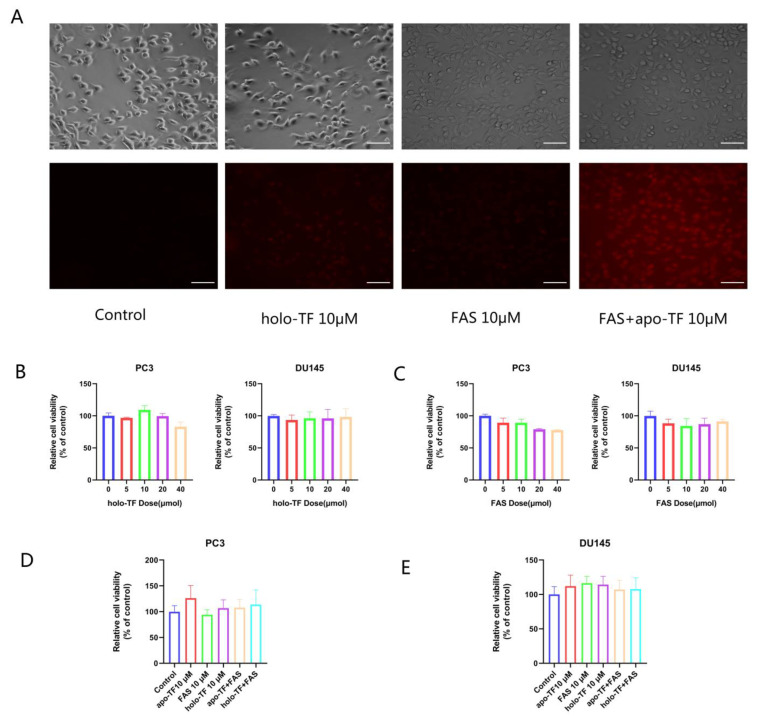
Intracellular iron levels increase after iron supplementation with the addition of holo-TF and FAS. (**A**) Cells were observed under a fluorescent microscope. Representative images of fluorescence staining after iron supplementation with 10 μM holo-TF and FAS with or without apo-TF in PC3 cells for 30 min. Scale bars: 100 μm. (**B**) Relative cell viability of PC3 and DU145 cells after different doses of holo-TF treatment for 3 h. (**C**) Relative cell viability of PC3 and DU145 cells after different doses of FAS treatment for 3 h. (**D**,**E**) Relative cell viability of PC3 and DU145 cells after apo-TF, holo-TF, FAS alone or in combination treatment for 3 h.

Using a ferrous ion fluorescent probe, our study confirmed that holo-TF and FAS supplementations increase intracellular iron concentration. After treatment with holo-TF (10 μM), FAS (10 μM) or FAS with apo-TF for 30 min, the fluorescence in PC3 cells increased, indicating an increase in intracellular labile iron, and apo-TF facilitated the FAS entry into cells (Figure 2A). The results of cell proliferation experiments showed that apo-TF (10 μM), holo-TF (10 μM) or FAS (10 μM) treatment alone or in combination did not cause cytotoxicity to PC3 and DU145 cells (Figure 2B–E).

### 3.3. Iron Supplementation Increases ROS Generation Induced by VC, Thereby Enhancing Cytotoxicity; Iron Chelators Protect Cancer Cells from the Toxicity Induced by VC

**Figure 3:**
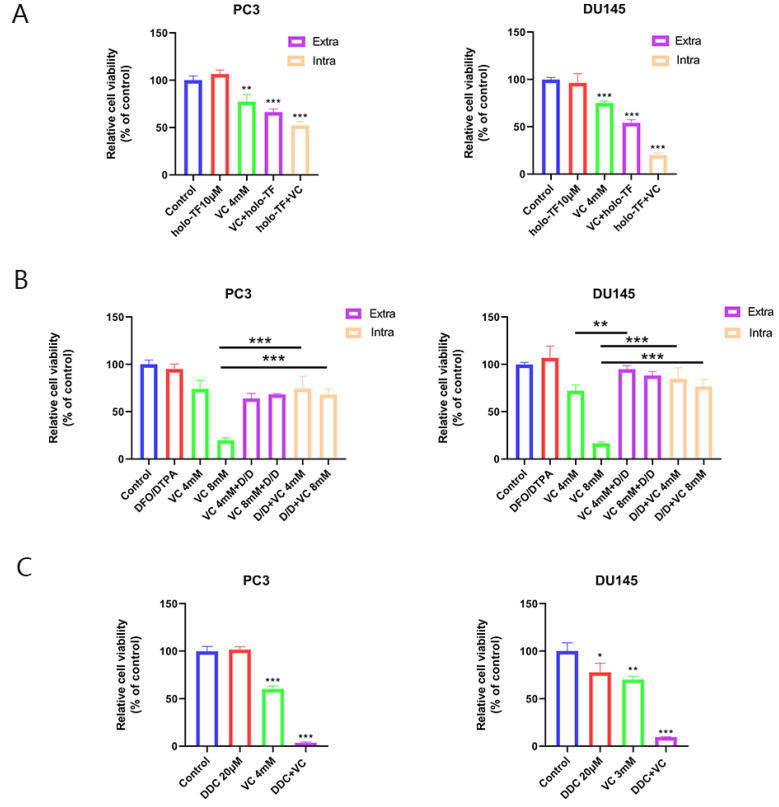
Iron supplementation enhances cytotoxicity induced by VC, which could be protected by iron chelators. (**A**) Relative cell viability of PC3 and DU145 cells after being pre-treated with 10 μM holo-TF for 3 h, washed with PBS and then treated with 4 mM VC for 3 h (iron supplementation intracellular) or co-exposure to 10 μM holo-TF and 4 mM VC for 3 h (iron supplementation extracellular). (**B**) PC3 and DU145 cells were pre-treated with 200 μM DFO and 1 mM DTPA (D/D) for 3 h followed by exposure for 3 h to 4/8 mM VC, or co-exposure to D/D and 4/8 mM VC for 3 h. (**C**) PC3 and DU145 cells were treated with different doses of VC alone or combined with 20 μM DDC for 3 h. * *p* < 0.05, ** *p* < 0.01, *** *p* < 0.001.

VC (4 mM) treatment for 3 h alone decreased survival to 77.37% and 75.10% in PC3 and DU145 cells, respectively. To elucidate the role of intracellular vs. extracellular iron on VC toxicity, PC3 and DU145 cells were pre-cultured with holo-TF (10 μM) for 3 h, and they were either washed with PBS or not washed prior to treatment with 4 mM VC for 3 additional h. Whether they were washed with PBS or not washed before the VC treatment, the survival of PC3 and DU145 cells further decreased compared with VC treatment alone (Figure 3A). Interestingly, pre-incubation with holo-TF before the VC treatment was more efficient in enhancing cytotoxicity than when added concomitantly, which suggested that intracellular iron content was a critical determinant of the anti-cancer effects of VC.

To further investigate the role of iron in VC-induced cytotoxicity, PC3 and DU145 cells were exposed to DFO/DTPA (DD) for 3 h, which was followed by 4/8 mM VC for 3 h. There was no difference in survival for cells treated with DFO/DTPA alone compared to the control. However, on exposure to VC (8 mM) alone, only 19.84% and 16.48% survived as assessed by cell proliferation assays (Figure 3B). After being pre-incubated for 3 h with iron chelators, DD significantly inhibited VC-induced toxicity in both PC3 and DU145 cells, and survival increased to 68.04% and 76.56%. The results suggest that iron chelators could partially eliminate VC-induced cytotoxicity.

After iron supplementation, there was a significant increase in ROS generation compared to the VC treatment alone (Figure 1B). Iron supplementation induces H_2_O_2_ generated by VC to further move toxic superoxide through the Fenton reaction. To verify the toxicity of superoxide, we used DDC, which is a superoxide dismutase (SOD) inhibitor that blocks the dissociation of O_2−_ into H_2_O_2_ [19]. As shown in Figure 3C, DDC significantly increased the cytotoxicity of VC to PC3 and DU145. The cell survival rate of VC combined with DDC decreased by 56.88% and 60.75% in PC3 and DU145 cells, respectively, compared with VC alone. These results suggest that the toxic effect of superoxide anion (O_2−_) on cells is higher than of H_2_O_2_.

### 3.4. The Synergistic Anti-Tumor Effect of Iron Supplementation and VC Is Related to TF

**Figure 4:**
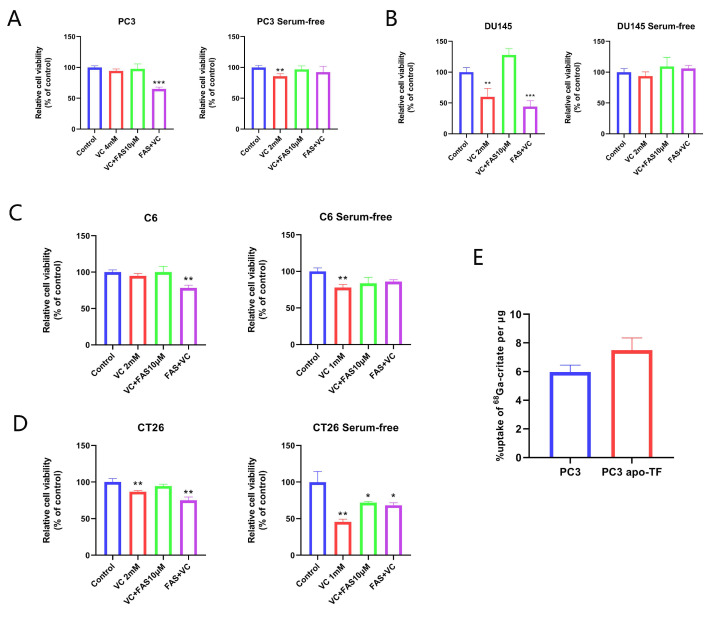
The effect of iron supplementation to VC-induced cytotoxicity is associated with TF. (**A**–**D**) Relative cell viability of four cell lines (PC3, DU145, C6 and CT26) after being pre-treated with 10 μM FAS for 3 h, washed with PBS and then treated with different doses of VC for 3 h (iron supplementation intracellular) or co-exposure to 10 μM FAS and different doses of VC for 3 h (iron supplementation extracellular). (**E**) The ^68^Ga-citrate uptake rate of PC3 cells with and without apo-TF for 1 h. * *p* < 0.05, ** *p* < 0.01, *** *p* < 0.001.

When FAS were used as the iron source, pre-culture with FAS enhanced cell killing by VC in PC3 cells. Interestingly, FAS do not enhance the anti-tumor effect of VC when there is concurrent exposure to PC3 and DU145 cells (Figure 4A,B). The same results were observed in C6 and CT26 cells: the cell survival rate was 94.94% and 86.83% when treated with 2 mM VC alone for 3 h; when treated with 10 μM FAS for 3 h and then with VC for 3 h, the cell survival rate decreased to 78.50% and 75.08%, respectively (Figure 4C,D). However, after being treated with FAS and VC for 3 h simultaneously, the cell survival rates were 100.11% and 94.67%, respectively. As shown in Figure 4, the simultaneous addition of FAS and VC did not reproduce the synergistic inhibition effect, and it even partially eliminated the cytotoxicity of VC alone. That is, the supplement of extracellular and extracellular iron has different effects on the cytotoxicity of VC. Holo-TF supplements iron that binds to transferrin, while FAS supplements iron that does not bind to transferrin. Thus, we speculate that the different effects of the two iron sources may be related to TF.

In order to exclude the interference of transferrin in serum, we used a serum-free medium in the proliferation experiment. The results show that FAS pre-culture after serum removal has no significant enhancement effect on the cytotoxicity of VC in all four cell lines (Figure 4A–D), which again confirms the effect of TF on the cytotoxicity of VC.

The results of the cell uptake experiment in vitro showed that the cellular uptake of ^68^Ga-citrate has a trend of increasing after adding apo-TF (*p* = 0.054). The ^68^Ga-citrate uptake rates of PC3 cells with and without apo-TF were 7.50% and 5.97%, respectively (Figure 4E). This result indicates that TF enhance ^68^Ga-citrate uptake in PC3 cells.

### 3.5. The Synergistic Anti-Tumor Effect of Iron Supplementation and VC Is Related to the TFR Expression of Tumor Cells

**Figure 5:**
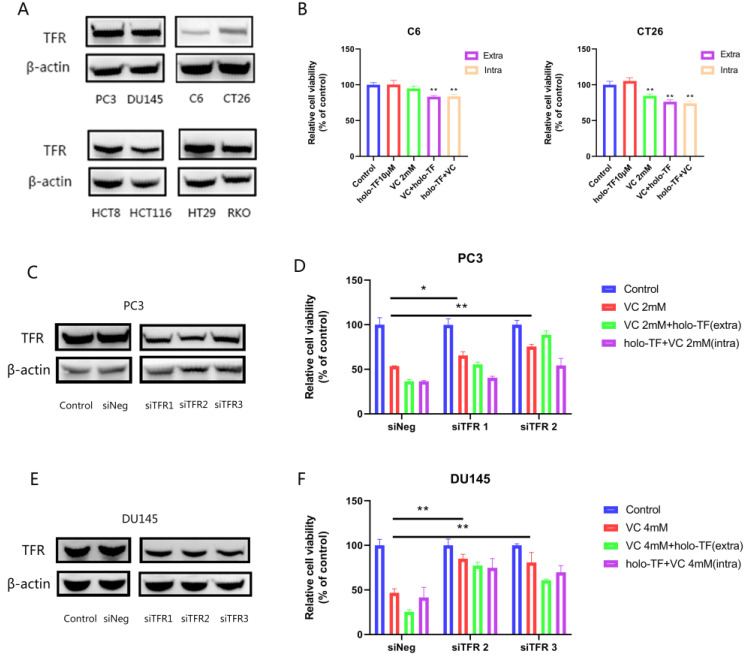
The influence of iron supplementation to VC-induced cytotoxicity is associated with TFR; TFR knockdown attenuates VC-induced cytotoxicity. (**A**) TFR expression of eight cell lines (PC3, DU145, C6, CT26, HCT8, HCT116, HT29 and RKO). (**B**) Relative cell viability of C6 and CT26 cells after being pre-treated with 10 μM holo-TF for 3 h, washed with PBS and then treated with 2 mM VC for 3 h (iron supplementation intracellular) or co-exposure to 10 μM holo-TF and 2 mM VC for 3 h (iron supplementation extracellular). (**C**,**E**) The TFR expression of PC3 and DU145 cells after TFR knockdown after 48 h verified by Western blotting. (**D**,**F**) Relative cell viability of PC3 and DU145 cells after being pre-treated with 10 μM holo-TF for 3 h, washed with PBS and then treated with different doses of VC for 3 h (iron supplementation intracellular) or co-exposure to 10 μM holo-TF and different doses of VC for 3 h (iron supplementation extracellular). The uncropped immunoblot images can be found in Figure S1. The IntDen of western blot bands can be found in Table S1. * *p* < 0.05, ** *p* < 0.01.

In order to further study whether there is a difference in the effect of TFR expression on the anti-tumor effect of VC, two cell lines with high TFR expression (DU145 and PC3) and two cell lines with low TFR expression (C6 and CT26) were selected according to the results of the Western blot analysis (Figure 5A). Cell proliferation experiments showed that iron supplementation in cell lines with high TFR expression (DU145 and PC3) increased the cytotoxicity of VC more significantly than the cell lines with low TFR expression (C6 and CT26) (Figure 3A and Figure 5B). Thus, we speculate that the different effects of the two iron sources may be related to TFR.

In fact, many cancer cells exhibit iron metabolism disorders, which is manifested by the up-regulation of several iron uptake pathways, such as TFR, and down-regulation of iron output and storage pathways [10]. Therefore, we used siRNA to knock down the expression of TFR in PC3 and DU145 cells to explore the importance of TFR in VC treatment.

The results presented in Figure 5C,E demonstrate a significant suppression of TFR expression after the transfection of siRNA (siTFR) after 48 h in PC3 and DU145 cells. Then, we randomly selected two siRNA sequences in subsequent experiments. Our results showed that TFR knockdown decreased the cytotoxicity of VC. After treatment with VC for 3 h, the PC3 cell survival rate of the negative control group (siNeg group) was 53.61%. The cell survival rate of the siTFR-1 group was 65.54% (*p* < 0.05; compared with the siNeg group). The cell survival rate of the siTFR-2 group was 75.41% (*p* < 0.01; compared with the siNeg group). After treatment with VC for 3 h, the DU145 cell survival rate of the negative control group (siNeg group) was 45.15%, and the cell survival rate of the siTFR-2 group was 88.10% (compared with the siNeg group; *p* < 0.01). The cell survival rate of the siTFR-3 group was 84.5% (compared with the siNeg group, *p* < 0.01). The results showed that the sensitivity of prostate cancer cells to VC therapy decreased after inhibiting the expression of TFR. The results also showed that after inhibiting the expression of TFR in PC3 and DU145 cells, the toxic enhancement effect of holo-TF on VC was weaker than that in the control group when both drugs were introduced simultaneously (Figure 5D,F).

### 3.6. PET-CT Imaging Experiments Showed That ^68^Ga-Citrate Uptake Was Associated with the TF/TFR System of Tumor Tissue

**Figure 6:**
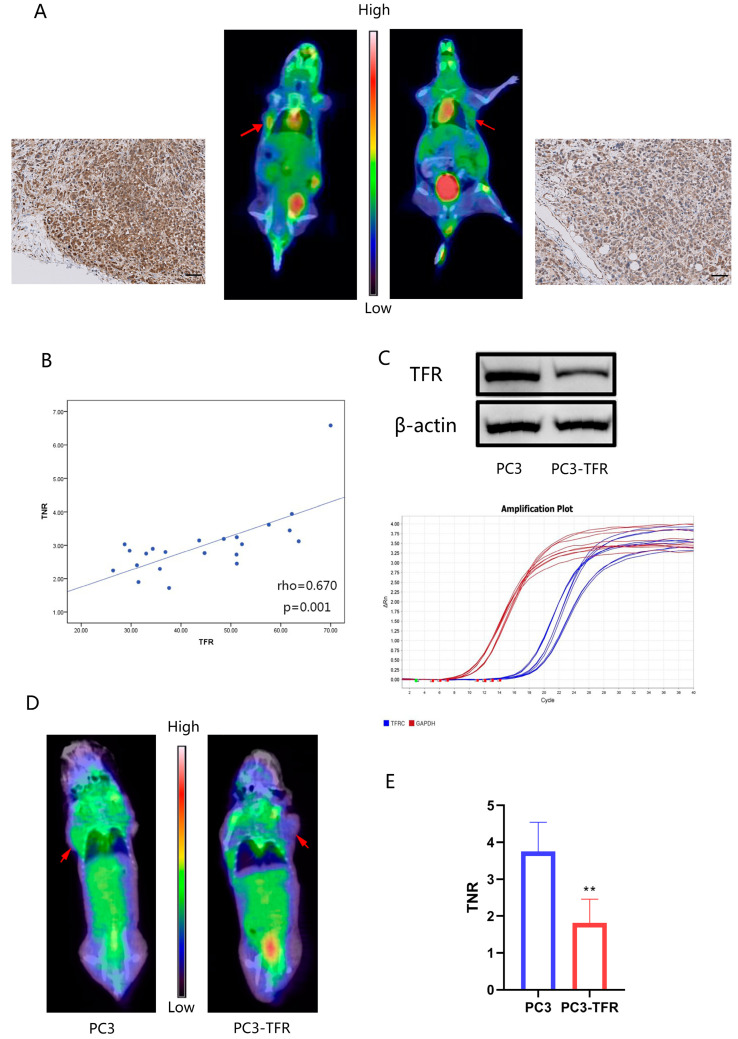
^68^Ga-citrate uptake of PC3 xenograft tumors is correlated with TFR expression. (**A**) ^68^Ga-citrate PET/CT images and TFR immunohistochemistry staining of two nude mice with PC3 xenograft tumors. Scale bars: 100 μm. (**B**) Scatter plot/correlation between TFR expression and TNR of PC3 xenograft tumors. Trend lines and Spearman’s rho correlation coefficient are shown. (**C**) Western blot and qPCR analyses of TFR expression in stably transfected PC3 cell lines. The uncropped immunoblot images can be found in Figure S1. The IntDen of western blot bands can be found in Table S1. (**D**,**E**) ^68^Ga-citrate PET/CT images of nude mice with PC3/stably transfected PC3 cell xenograft tumors. ** *p* < 0.01.

The results of PET-CT imaging and immunohistochemistry staining showed that positive TFR staining in the tumor was associated with a high uptake of ^68^Ga-citrate. ^68^Ga-citrate showed high uptake (TNR = 3.94) in the tumor with high expression TFR and low uptake (TNR = 1.90) in the tumor with low expression TFR (Figure 6A). The statistical results showed that the positive expression of TFR staining was positively correlated with the uptake of ^68^Ga-citrate in tumors (*p* < 0.01) (Figure 6B). To further verify the correlation between TFR expression and ^68^Ga-citrate uptake, we obtained a stably transfected PC3 cell line with TFR knockdown and constructed a corresponding xenograft tumor mouse model (Figure 6C). PET-CT imaging showed that ^68^Ga-citrate uptake in tumors was significantly reduced in the TFR knockdown tumor (*p* < 0.01) (Figure 6D,E).

## 4. Discussion

Iron is a trace element that is necessary for basic functions, and malignant cancer cells have a high requirement for iron. High levels of ferritin have been detected in the plasma of many malignant tumors, which are associated with poor patient outcomes [20,21]. Generally, iron is internalized by endocytosis via the TF/TFR system of cells [22,23], and cancer cells can maintain a cellular iron balance by regulating the TF/TFR system [24]. Tumor cells raise intracellular iron concentrations through the up-regulation of iron metabolic proteins, such as TFR-1, DMT1 and hepcidin [25,26,27]. Using FeRhoNox-1™ to measure intracellular labile iron demonstrated that holo-TF elevated the intracellular iron content in the two cell lines. FAS mildly elevates the intracellular iron content without serum, and apo-TF could promote FAS’ entry into cells. Thus, we consider that the TF/TFR-mediated pathway is the major iron uptake pathway for cells.

At present, most studies have confirmed that the anti-tumor effect of high-dose VC is mainly based on its ability to generate excessive H_2_O_2_ in tumor cells, which induce the apoptosis, pyknosis and necrosis of cancer cells [2,6]. The specific mechanism is related to the self-oxidation of VC; that is, VC reacts with oxygen (O_2_) to produce ROS. However, VC auto-oxidation requires the donation of an electron to redox-active transition metal ions, such as by reducing ferric (Fe^3+^) ions to ferrous (Fe^2+^) ions. Iron ion is an important participant in intracellular redox reaction. According to the Fenton reaction, H_2_O_2_ reacts with Fe^2+^ to form hydroxyl radicals, which have greater toxicity than H_2_O_2_ [28]. Previous studies have suggested that VC-induced cytotoxicity was associated with ferritin concentrations in cancer cells [29,30]. Iron chelators (DFO and DTPA) could inhibit the redox cycling of iron [31] and decline intracellular labile iron’s catalytic activity [32], thereby protecting cells from Fenton reaction-induced damage [33]. 

Our results support the hypothesis that the anti-tumor effects of pharmacological VC in prostate cancer are mediated by ROS and redox active iron. Our results also suggest that increased intracellular labile iron enhances VC-induced cytotoxicity. Increasing the LIP by supplying Fe^2+^, as holo-TF or FAS, sensitized prostate cancer cells to VC. Our finding seems contradictory to previous studies, which found that supplementing Fe^2+^ can actually protect cells from H_2_O_2_ [34,35]. However, both of these previous studies used iron unbounded transferrin to supplement iron, which are ferrous sulfate and ferrous ammonium sulfate, respectively. In normal physiological conditions, iron is tightly bound to transferrin in circulation [36]. However, iron is catalytically active only in the unbound state [6]. 

In this study, we found that unbound iron should not be given simultaneously with VC but rather in advance of VC to ensure that the iron actually enters the intracellular space, thereby exerting its potentiating effect on VC-induced cytotoxicity. Our results support the hypothesis that the combination of H_2_O_2_ and intracellular redox-active metal ions is important for VC to inhibit cancer cell proliferation [29]. HO· is the primary oxidant generated by the Fenton reaction [37]. However, HO· is so reactive that the diffusion distance is only about 6 nm in cells [38]. HO· generated by the Fenton reaction in the media is largely free of harm to cells, because it will react with media components but not cells [5]. This is consistent with previous results, which show that iron can react with extracellular H_2_O_2_, thereby eliminating the cytotoxicity of VC to cancer cells [39,40]. This also explains why simultaneously adding FAS could reverse the cytotoxicity of VC but holo-TF cannot, as per our experiments. FAS supplements iron that does not bind to transferrin, which immediately reacts with extracellular H_2_O_2_ to eliminate its cytotoxicity. Thus, increasing intracellular iron enhanced VC-induced cytotoxicity rather than extracellular iron. Furthermore, as a strong reductant, VC reduces trivalent iron to divalent iron and then restarts the Fenton reaction and kills tumor cells [41], which may be another important reason for the synergistic effect of VC and iron.

In this study, we explored the relationship between Ga-68 citrate scanning (Ga-68 scan) and immunohistochemistry TFR expression in nude mice for PC3 xenografts. We found that xenograft tissues with a high ^68^Ga-citrate uptake were significantly more likely to have a higher TFR expression. This could be explained by the mechanism by which citrate initially combines with transferrin in the blood, and then, the complex is incorporated into cells through TFR [42,43]. The only difference is that the previous study used Ga-67, whereas we used Ga-68. Ga-68 is an isotope of Ga-67 with similar physicochemical properties. However, Ga-68 has a shorter half-life than Ga-67, and it is easier to obtain by a ^68^Ge/^68^Ga generator than Ga-67 is by an accelerator [44]. Thus, using ^68^Ga-labeled citrate in PET/CT imaging has social and economic benefits. We used preclinical tumor models to confirm that ^68^Ga-citrate uptake is dependent on TFR expression and activity, which is consist with previous findings that ^68^Ga-citrate could bind to apo-TF in blood as an Fe (III) biomimetic [18]. Thus, it may be reliable to predict the anti-tumor efficacy of VC combined with iron supplementation by Ga-68 scanning results. According to the results of the cellular experiments in our study, cells with high TFR expression were more sensitive to VC combined with iron supplementation treatment.

## 5. Limitations of the Study

In our study, the effects of TFR expression to VC treatment combined with iron supplementation were mainly detected by experiments in vitro, which are yet to be validated in animal experiments. This is a further research direction for us.

## 6. Conclusions

Intracellular iron supplementation selectively sensitizes cancer cells to high-dose VC by disrupting the oxidative metabolism, and we consider that ^68^Ga-citrate PET imaging may be able to screen out tumor patients with high TFR expression, which could be more sensitive to VC treatment combined with iron supplementation.

## Data Availability

The data presented in this study are available in this article and supplementary material.

## Supplementary Materials

The following supporting information can be downloaded at: https://www.mdpi.com/article/10.3390/cancers14184507/s1, Figure S1: The western blot original figures (uncropped blots); Table S1: The IntDen of western blot bands.
