# Supplementary material for: Role of Fe, Transferrin and Transferrin Receptor in Anti-Tumor Effect of Vitamin C
Source: Cancers (Basel). 2022 Sep 17;14(18):4507. doi: 10.3390/cancers14184507 (PMC9496724; doi:10.3390/cancers14184507)
Supplement: Supplementary file 1 [file cancers-14-04507-s001.zip › cancers-1892325-supplementary.pdf]

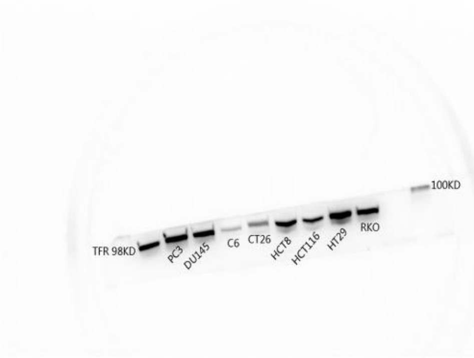

figure 5A

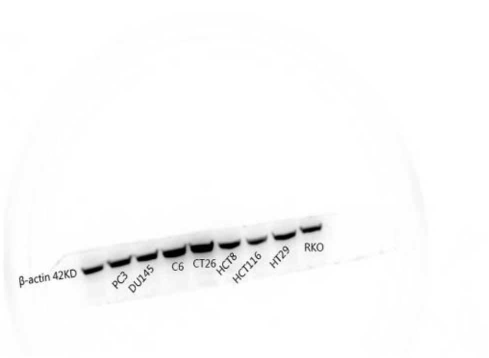

figure 5A

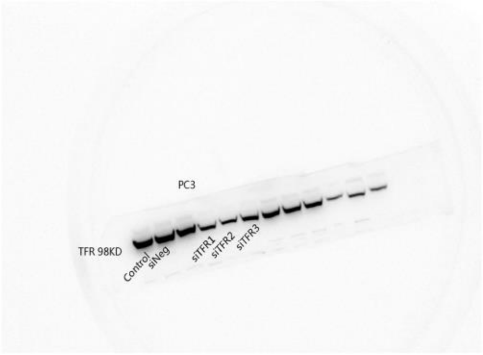

figure 5C

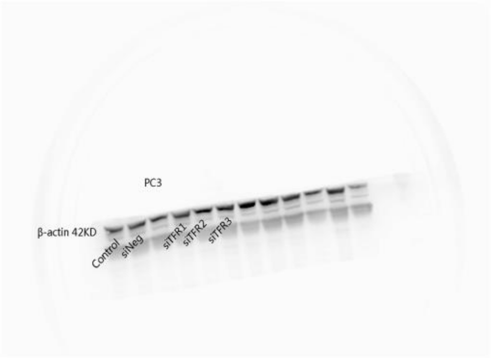

figure 5C

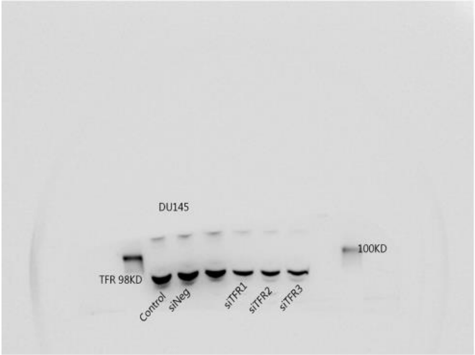

figure 5E

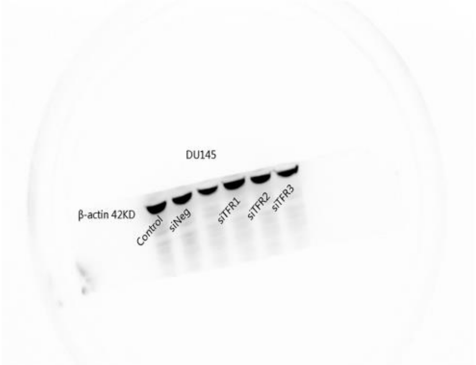

figure 5E

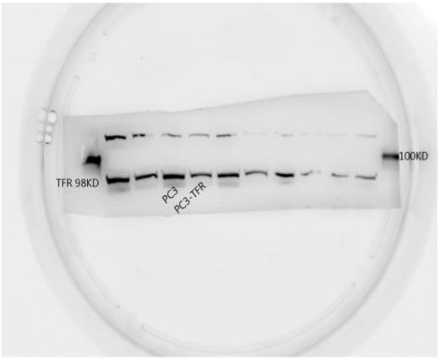

figure 6C

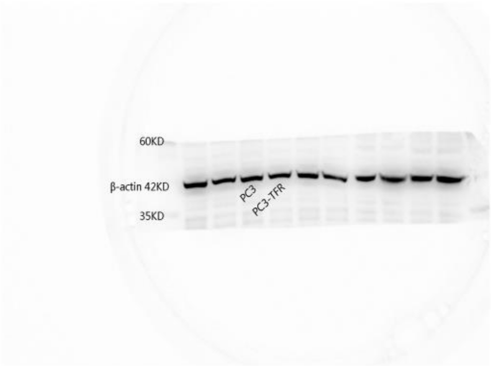

figure 6C

Figure S1. The western blot original figures (uncropped blots).

Figure 5A								
	PC3	C6	CT26	DU145	HCT8	HCT116	HT29	RKO
TFR	245351	23867	56666	202889	173732	98795	251467	193141
β-actin	205132	316581	369465	209597	179288	132384	192658	134265
Figure 5C								
PC3	Control	siNeg	siTFR-1	siTFR-2	siTFR-3			
TFR	288939	271746	103238	84197	138672			
β-actin	75882	72744	79378	80023	91565			
Figure 5E								
DU145	Control	siNeg	siTFR-1	siTFR-2	siTFR-3			
TFR	299637	296788	146319	143416	120215			
β-actin	318279	293911	311022	277413	224228			
Figure 6C								
	PC3	PC3-siTFR						
TFR	208819	80934						
β-actin	194932	157595						

Table S1. The IntDen of western blot bands.
